# Molecular Basis of a Dominant T Cell Response to an HIV Reverse Transcriptase 8-mer Epitope Presented by the Protective Allele HLA-B*51:01

**DOI:** 10.4049/jimmunol.1302667

**Published:** 2014-03-05

**Authors:** Chihiro Motozono, Nozomi Kuse, Xiaoming Sun, Pierre J. Rizkallah, Anna Fuller, Shinichi Oka, David K. Cole, Andrew K. Sewell, Masafumi Takiguchi

**Affiliations:** * Cardiff University School of Medicine, Heath Park CF14 4XN, United Kingdom;; † Center for AIDS Research, Kumamoto University, Chuo-ku, Kumamoto 860-0811, Japan; and; ‡ AIDS Clinical Center, National Center for Global Health and Medicine, Shinjuku-ku, Tokyo 162-8655, Japan

## Abstract

CD8^+^ CTL responses directed toward the HLA-B*51:01–restricted HIV-RT_128–135_ epitope TAFTIPSI (TI8) are associated with long-term nonprogression to AIDS. Clonotypic analysis of responses to B51-TI8 revealed a public clonotype using TRAV17/TRBV7-3 TCR genes in six out of seven HLA-B*51:01^+^ patients. Structural analysis of a TRAV17/TRBV7-3 TCR in complex with HLA–B51-TI8, to our knowledge the first human TCR complexed with an 8-mer peptide, explained this bias, as the unique combination of residues encoded by these genes was central to the interaction. The relatively featureless peptide-MHC (pMHC) was mainly recognized by the TCR CDR1 and CDR2 loops in an MHC-centric manner. A highly conserved residue Arg^97^ in the CDR3α loop played a major role in recognition of peptide and MHC to form a stabilizing ball-and-socket interaction with the MHC and peptide, contributing to the selection of the public TCR clonotype. Surface plasmon resonance equilibrium binding analysis showed the low affinity of this public TCR is in accordance with the only other 8-mer interaction studied to date (murine 2C TCR–H-2K^b^-dEV8). Like pMHC class II complexes, 8-mer peptides do not protrude out the MHC class I binding groove like those of longer peptides. The accumulated evidence suggests that weak affinity might be a common characteristic of TCR binding to featureless pMHC landscapes.

## Introduction

The cytotoxic payload delivered by CD8^+^ CTLs destroys cells posing a threat to host health. To ensure that this cytotoxicity is specifically targeted toward aberrant cells, CTLs express a TCR that can distinguish between self- and non–self-peptides (usually ranging from 8–13 aa) presented on the surface of most nucleated cells in peptide–MHC class I (pMHCI) (1, 2). Thus, T cells have the ability to scan the cellular proteome via the cell surface, providing an important mechanism for targeting diseased cells. CTLs constitute our main defense against intracellular infections and can destroy virally infected cells. It is well established that certain HLA molecules, such as HLA-B*27, HLA-B*51, and HLA-B*57, are associated with better control of HIV infection (3–5). These findings attest to the importance of CD8^+^ CTL responses in HIV infection and have generated considerable interest in the mechanisms behind this protection in HIV-1–infected individuals carrying these HLA alleles. In this study, we focused on the response to HLA-B*51:01–restricted HIV-RT_128–135_ 8-mer epitope (TAFTIPSI [TI8]). HLA-B*51:01 is associated with slow progression to AIDS, and B51-TI8–specific T cells strongly suppress HIV-1 replication in vitro (6). Furthermore, the magnitude of the B51-TI8–specific CD8^+^ T cell response was significantly correlated to low plasma viral load in chronically HIV-1–infected HLA-B*51:01^+^ Japanese hemophiliacs, whereas no correlation was found between the magnitude of the CD8^+^ T cell response to three other dominant HLA-B51–restricted HIV-1 epitopes (7). Collectively, these correlative data suggest that the response to B51-TI8–specific CD8^+^ T cells might play an important role in the control of HIV-1 replication.

We undertook to dissect HLA-B*51:01–restricted CTL responses to this important epitope by examining the TCRs raised against the B51-TI8 epitope in responding patients. We identified a public CTL clone (3B) expressing a TCR encoded by TRAV17/TRBV7-3 TCR genes, specific for B51-TI8. We solved the structure of the 3B TCR in complex B51-TI8 and conducted a biophysical analysis of the interaction. To our knowledge, the 3B–B51-TI8 structure is the first human TCR complex with HLA-B*51:01 and the first containing a short 8-mer peptide without a prominent central bulge. Our data reveal the genetic and molecular mechanism explaining the predominance of TRAV17/TRBV7-3 TCRs in HLA-B*51:01^+^ HIV^+^ patients and provide new structural insights into CTL recognition of a flat 8-mer peptide presented by HLA-B*51:01.

## Materials and Methods

### Patients

Seven chronically HIV-1–infected Japanese individuals were recruited for the current study, which was approved by the ethics committees of Kumamoto University and the National Center for Global Health and Medicine, Japan. Written informed consent was obtained from all subjects according to the Declaration of Helsinki. We focused on seven HLA-B*51:01^+^ Japanese individuals chronically infected with HIV-1 because HLA-B*51:01–restricted TI8-specific CTLs were induced by stimulating PBMCs from only these individuals with TI8 peptides. Three individuals (KI-021, KI-051, and KI-124) are hemophiliacs and long-term nonprogressors. Clinical records and HLA type of these individuals are shown in Supplemental Table I.

### Generation of TI8-specific CTLs

HLA-B*51:01–restricted TI8-specific CTL clones were generated from HIV-1–specific bulk-cultured T cells established from seven HLA-B*51:01^+^ Japanese individuals chronically infected with HIV-1 by limiting dilution in U-bottom 96-well microtiter plates (Nunc, Roskilde, Denmark). Each well contained 200 μl cloning mixture (∼1 × 10^6^ irradiated allogeneic PBMCs from healthy donors and 1 × 10^5^ irradiated C1R-B*51:01 cells prepulsed with the corresponding peptide at 1 μM in RPMI 1640 supplemented with 10% human plasma and 200 U/ml human rIL-2).

### TCR clonotype analysis

HLA-B*51:01–TI8 tetramers were generated as previously described (7). CTL clones were stained with the tetramers, anti-CD8 mAb, and 7-aminoactinomycin D (7-AAD), and then tetramer^+^CD8^+^7-AAD^−^ cells were sorted by using an FACSAria I (BD Biosciences). The cryopreserved PBMCs from four chronically HIV-1–infected HLA-B*51:01^+^ individuals were stained with B51-TI8 tetramers, anti-CD8 mAb, and 7-AAD. HLA-B*51:01–TI8 tetramer^+^ CD8^+^ 7-AAD^−^ cells were sorted into 96-well plates (Bio-Rad) by using an FACSAria I (BD Biosciences). For samples from sorted single HLA-B*51:01–TI8-specific CD8^+^ T cells and CTL clones, unbiased identification of TCR αβ-chain usage was assessed as previously described (8). A modification to the protocol was applied by using illustra ExoStar (GE Healthcare), which contains alkaline phosphatase and exonuclease 1 to remove unincorporated primers and nucleotides from the amplification reaction prior to subsequent steps. For bulk-sorted HLA-B*51:01–TI8-specific CD8^+^ T cells, TCR genes were cloned with a Zero Blunt TOPO PCR cloning kit (Invitrogen), and then several clones were sequenced. Sequencing was done with a BigDye Terminator v3.1. cycle sequencing kit (Applied Biosystems) and analyzed by ABI 3500 and 3500xL Genetic Analyzer (Applied Biosystems, Carlsbad, CA). All TCR genes are identified using the ImMunoGeneTics database (http://www.imgt.org).

### Protein expression and purification

The extracellular domains of 3B TCR α-chain (residues 1–207), 3B TCR β-chain (residues 1–247), and B51 H chain (residues 1–248 ± a biotinylation tag) were inserted into separate pGMT7 expression plasmids for expression under the T7 promoter (9). β_2_-microglobulin (residues 1–247) was similarly expressed. Competent Rosetta DE3 *Escherichia coli* cells were used to produce proteins using 0.5 mM isopropyl β-d-thiogalactoside to induce expression as described previously (9–11). Biotinylated pMHCI was prepared as previously described (12). TCR sequences were engineered to incorporate a nonnatural disulphide bond to aid heterodimerization (9, 13).

### Surface plasmon resonance experiments

Surface plasmon resonance (SPR) equilibrium binding analysis was performed using a BIAcore3000 (GE Healthcare Life Sciences) equipped with a CM5 sensor chip as previously reported (12, 14, 15). HLA-A*02:01–ALWGPDPAAA and HLA-B*35:01–VPLRPMTY were used as a negative controls on flow cells 1 and 3 in three separate experiments. SPR equilibrium analyses were carried out to determine the *K*_D_ values for the TCR at 25°C in triplicate (representative data shown). For all experiments, ∼300 response units pMHC was coupled to the CM5 sensor chip surface. The TCR was then injected at concentrations ranging from 10 times above and 10 times below the known *K*_D_ of the interaction at 45 μl/min. The *K*_D_ values were calculated assuming 1:1 Langmuir binding (AB = B*AB_MAX_/[*K*_D_ + B]), and the data were analyzed using a global fit algorithm (BIAevaluation 3.1).

### Crystallization, diffraction data collection, and model refinement

All protein crystals were grown at 18°C by vapor diffusion via the sitting drop technique. A total of 200 nl 1:1 molar ratio TCR and pMHCI (10 mg/ml) in crystallization buffer (10 mM TRIS [pH 8.1] and 10 mM NaCl) was added to 200 nl reservoir solution. 3B–B51-TI8 crystals were grown in 0.2 M sodium sulfate, 0.1 M BisTris propane (pH 6.5), and 20% w/v PEG 3350 (16). Data were collected at 100 K at the Diamond Light Source (Oxfordshire, U.K.). All datasets were collected at a wavelength of 0.976 Å using an ADSC Q315 CCD detector (Area Detector Systems Corporation). Reflection intensities were estimated with the XIA2 package (17), and the data were scaled, reduced, and analyzed with SCALA and the CCP4 package (18). Structures were solved with molecular replacement using PHASER (19). Sequences were adjusted with COOT (20) and the models refined with REFMAC5. Graphical representations were prepared with PyMOL (21). Data reduction and refinement statistics are shown in Table I. The reflection data and final model coordinates were deposited with the Protein Data Bank database (http://www.rcsb.org) (Protein Data Bank 4MJI).

## Results

### A public TRAV17/TRBV7-3–encoded TCR dominates responses to B51-TI8

**FIGURE 1.**
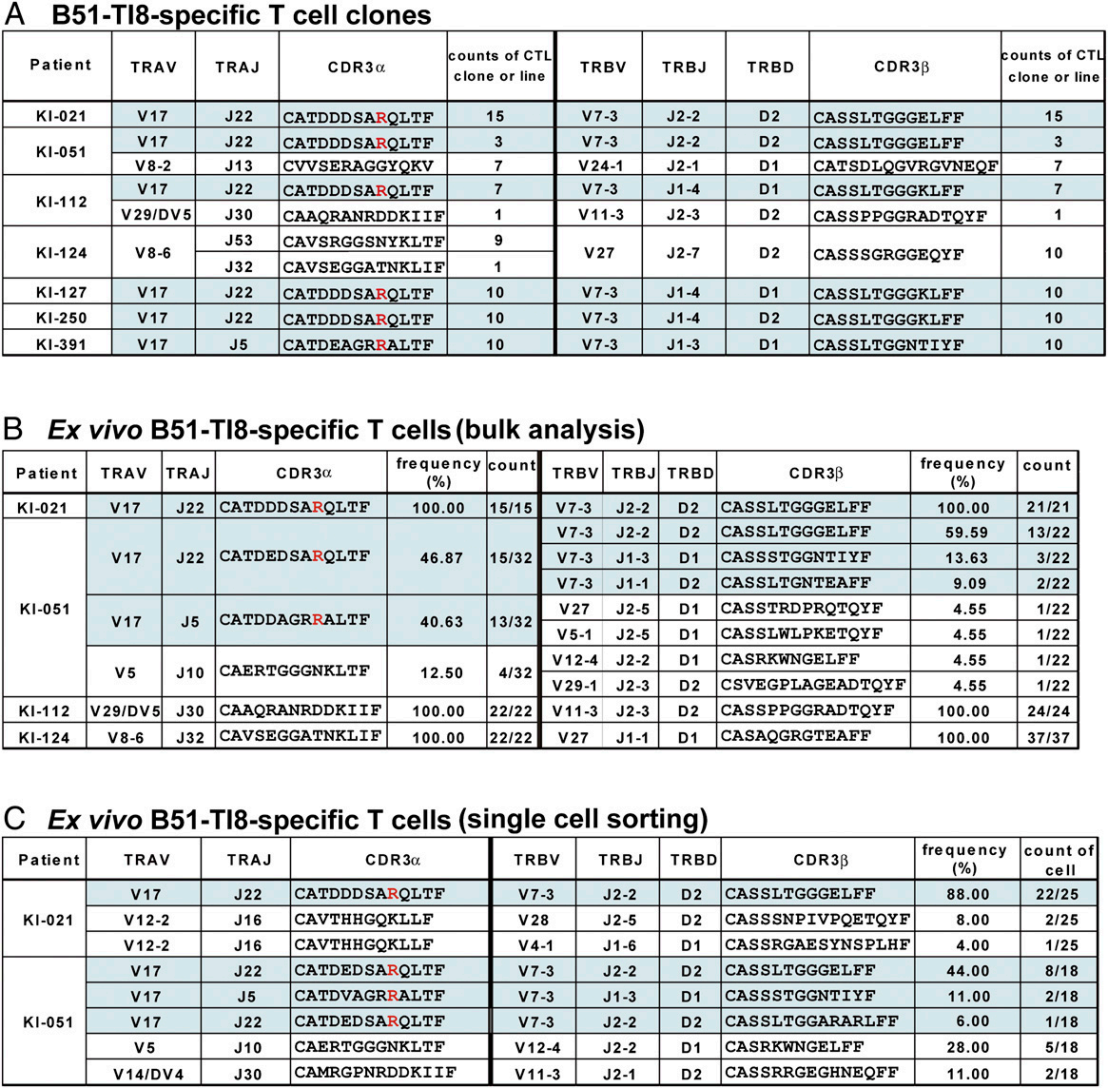
Detection of B51-TI8–specific CTLs expressing public TCRs with the TRAV17/TRBV7-3 genes. (**A**) TCR αβ-chain usage of B51-TI8–specific CTL clones established from seven HLA-B*51:01^+^ individuals infected with HIV-1. (**B**) Bulk TCR sequence analysis of ex vivo HLA-B*51:01–TI8 tetramer binding CD8^+^ T cell populations from four of the individuals shown in (A). (**C**) TCR analysis of sorted single cells of ex vivo tetramer binding CD8^+^ T cells from two patients. TCRs with the TRAV17/TRBV7-3 genes are highlighted in blue. The conserved TCR CDR3α Arg^97^ residue is shown in red.

We first generated TI8-specific CTL clones from seven HLA-B*51:01^+^HIV^+^ patients who responded to this epitope. The staining of representative clones by pMHC tetramer with this B51-TI8 epitope is shown in Supplemental Fig. 1. A total of 73 clones were analyzed for their TCRα- and TCRβ-chain sequences by previously described methodology (8). In patients KI-021, KI-127, KI-250, and KI-391, only T cells expressing TCRs with TRAV17/TRBV7-3 genes were detected (Fig. 1A). Patients KI-051 and KI-112 generated CTL clones expressing TRAV17/TRBV7-3 TCRs, but also expressed a further private B51-TI8–specific clonotype. The remaining patient (KI-124) used a completely different TCR made from the TRAV8-6 and TRBV27 genes. Thus, the TRAV17/TRBV7-3 clonotype was exclusively or predominantly detected in six out of seven individuals tested. Importantly, in five of these patients (except KI-391), the TCRα-chain consisted of a TRAV17, TRAJ22, and an identical CDR3 sequence: CATDDDSARQLTF. Curiously, the TCRβ-chain was encoded by a combination of the TRBV7-3, TRBJ2-2, and TRBD2 genes, with CDR3 sequence CASSLTGGELFF, in patients KI-021 and KI-051 (termed the 3B TCR in this study), whereas the TRBJ1-4 and TRBD1 or D2 were used in conjunction with TRBV7-3 in patients KI-112, KI-127, and KI-250 to produce the CDR3 sequence CASSLTGGKLFF (underlining indicates the only amino acid variation between these differentially encoded chains). This convergent evolution suggests that TCRs with this chain may have a strong selective advantage in vivo. A further patient, KI-391, exclusively used a different TCR made from a combination of the TRAV17/TRBJ5 and TRBV7-3/TRBJ1-3 genes. Although the exact sequence of both CDR3 loops differed in KI-391, these loops exhibited an identical length to that seen in all of the other patients (Fig. 1A). In summary, six out of seven patients used the TRAV17/TRBV7-3 TCR (three exclusively).

**Table I.** Summary of 3B–B51-TI8 cocomplex structure

	3B–B51-TI8
Hydrogen bonds (≤3.2 Å)	14
Hydrogen bonds (≤3.4 Å)	4
Vdw (≤3.5 Å)	37
Vdw (≤4 Å)	101
Total contacts	156
No. of α-chain CDR1/CDR2/CDR3 contacts (≤4 Å)	28/19/46
No. of β-chain CDR1/CDR2/CDR3 contacts (≤4 Å)	9/41/13
Peptide contacts	33
MHC contacts	123
Crossing angle	43.3°
BSA (TCR–MHC) (Å^2^)	2041.2
BSA (TCR–peptide) (Å^2^)	492.2
BSA (TCR–pMHC) (Å^2^)	2533.4
Surface complementarity (TCR–MHC)	0.6675
Surface complementarity (TCR–peptide)	0.6675
Surface complementarity (TCR–pMHC)	0.647

Examination of T cell clones, as above, could possibly introduce bias due to the ability of only certain clonotypes to proliferate in culture. To control for such potential distortion, we analyzed the clonotype of ex vivo B51-TI8–specific CD8^+^ T cells from four patients (KI-021, KI-051, KI-112, and KI-124) in whom we could readily gain PBMC samples and sort populations of B51-TI8 tetramer^+^ CD8^+^ T cells directly ex vivo. The clonotype of bulk-sorted B51-TI8–specific CD8^+^ T cells was analyzed for these four patients (Fig. 1B). In accordance with the T cell clone data, only T cells expressing TCRs with TRAV17/TRBV7-3 genes were detected in KI-021. Moreover, TRAV17/TRBV7-3 clonotypes with identical lengths of CDR3α and β loops were also predominantly detected in KI-051. In KI-112, only the TRAV29/DV5/TRBV11-3 clonotype was detected (Fig. 1B), although this clonotype was only expressed in one of eight CTL clones (Fig. 1A). Interestingly, only the TRAV8-6/TRBV27 clonotype was found in all CTL clones (Fig. 1A) and in ex vivo bulk-sorted T cells (Fig. 1B) from KI-124, but different TRAJ, TRBJ, and TRBD were found between two different cell sources. We further performed a single-cell TCR analysis for the samples from KI-021 and KI-051. Paired TCR usage data are shown in Fig. 1C. The TRAV17/TRAJ22/TRBV7-3/TRBJ2-2 clonotype was detected in 22 out of 25 B51-TI8–specific CD8^+^ T cells sorted from KI-021 PBMCs and 8 out of the 18 CD8^+^ T cells sorted from KI-051 PBMCs. Interestingly, there was an amino acid difference (Asp-Glu) at position 93 in CDR3α between KI-021 and KI-051 in ex vivo samples but not in CTL clones. TCR analysis for the bulk-cultured T cells from KI-051 used for CTL cloning demonstrated the same TCR sequence as the CTL clones (data not shown), indicating that CTLs carrying the TCR with Asp at position 93 were selected during in vitro stimulation. Thus, TRAV17/TRBV7-3 clonotype was also detected in ex vivo samples from at least two LTNPs (KI-021 and KI-051). These results show that the majority of B51-TI8–specific CTLs express a public TCR with type IV bias, consisting of a conserved TRBV or TRAV gene, TRBJ or TRAJ gene usage, and only one or two residue differences within the CDR3 loop in multiple individuals (22, 23). The public nature of this TCR the dominance of TI8 responses in HLA-B*51:01^+^ individuals, and the better disease prognosis associated with this allele combine to make the public response especially interesting.

### The 3B TCR binds to B51-TI8 with very weak affinity

**FIGURE 2.**
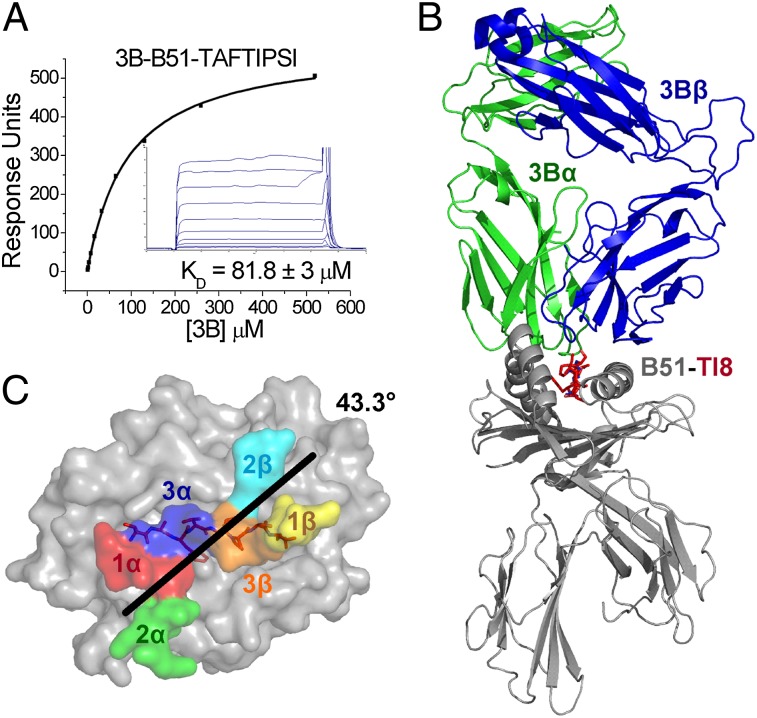
The 3B TCR binds to B51-TI8 with very weak affinity and engages B51-TI8 in classical orientation. (**A**) 3B TCR binding to B51-TI8. Ten serial dilutions of 3B TCR were measured in three separate experiments (with different protein preparations). Representative data from these experiments are plotted. The equilibrium binding constant (*K*_D_) values were calculated using a nonlinear curve fit (*y* = [P_1_*x*]/P_2_ + *x*). Mean plus SD values are shown. To calculate each response, 3B TCR was also injected over a control sample (HLA-A*02:01 in complex with ALWGPDPAAA peptide or HLA-B*35:01 in complex with VPLRPMTY peptide) that was deducted from the experimental data. (**B**) Overall binding mode of the 3B TCR (α-chain in green and β-chain in blue) interacting with B51 (gray) TI8 (red sticks). (**C**) Position and crossing angle of the 3B TCR CDR loops (CDR1α, red; 2α, green; 3α, blue; 1β, yellow; 2β, cyan; 3β, orange) over the B51 (gray surface)-TI8 (red sticks).

To explore the molecular mechanisms underlying the selective T cell response to B51-TI8, we generated a soluble form of the public TCR (3B) isolated from a CTL clone expressing the TRAV17/TRBV7-3 genes and the common CDR3 loops we detected in our clonotypic analysis (Fig. 1). The affinity of most pathogen-specific TCR–pMHCI interaction lies in the region of *K*_D_ = 1–10 μM (24). We used SPR to determine the equilibrium binding affinity of the 3B TCR for B51-TI8. The 3B TCR bound to B51-TI8 with the weakest affinity ever measured for a pathogenic epitope (24), *K*_D_ ≈81.8 μM, with kinetics that were too rapid to measure (Fig. 2A). This unusually weak affinity warranted further structure examination of Ag recognition by the public 3B TCR.

### The 3B TCR engages B51-TI8 in classical orientation

We solved the structure of the 3B–B51-TI8 complex to gain an atomic perspective on the molecular basis for the selection of this public clonotype in response to B51-TI8. The complex was solved to 2.99 Å, and molecular replacement was successful only in space group P1 with statistics consistent with the resolution (Supplemental Table II). Overall, the 3B TCR bound canonically, with the TCR CDRs contacting the pMHC surface (Fig. 2B) in a diagonal docking geometry (43.4°) (Fig. 2C) as previously reported for other TCR–pMHC complexes (25). The 3B TCR broadly contacted the MHC surface (123 total contacts with 22 different MHC residues) with only 33 contacts made between the TCR and peptide (Table I). The contact interface between the 3B TCR and B51-TI8 generated an overall buried surface area (BSA) of 2041.2 Å^2^ toward the higher end of the observed range for natural TCR–pMHC interactions (25).

### Interactions between the 3B TCR and the restriction triad dominate MHC contacts

**FIGURE 3.**
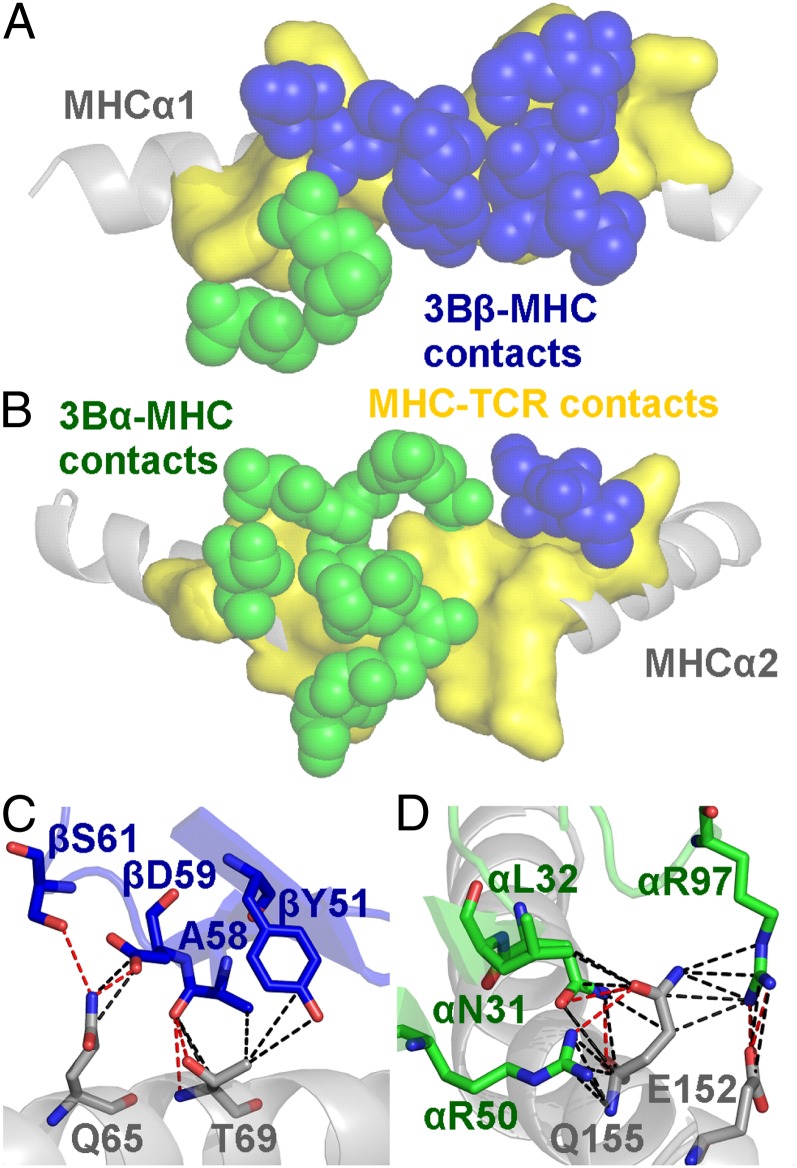
Interactions between the 3B TCR and the restriction triad dominate MHC contacts. (**A**) Position of the 3B TCR (α in green spheres and β in blue spheres) over the B51 α1 helix (residues contacted by the 3B TCR on the B51 α1 helix shown in yellow surface). (**B**) Position of the 3B TCR (α in green spheres and β in blue spheres) over the B51 α2 helix (residues contacted by the 3B TCR on the B51 α2 helix shown in yellow surface). (**C**) Contacts between the 3B TCR CDR2β loop and the MHCα1 domain including the restriction triad residues Q65 and T69. (**D**) Contacts between the 3B TCR CDR1 and 2α loops and the MHCα2 domain including the restriction triad residue Q155. These important stabilizing interactions include a number of Vdw contacts (black dotted lines, 4 Å cutoff) and hydrogen bonds (red dotted lines, 3.4 Å cutoff) with the MHC surface.

Previous structures of TCR–pMHC complexes have shown that most TCRs use three conserved MHC contact points (positions 65, 69, and 155; the restriction triad) (26, 27). The 3B TCR made a broad contact footprint with the MHC surface, contacting 22 different residues (Fig. 3A, 3B, Supplemental Table III). However, of the 123 interactions between the 3B TCR and the MHC surface, ∼40% (49 contacts) were with the restriction triad residues (Gln^65^, Thr^69^, and Gln^155^), including 6 out of 14 hydrogen bonds (Fig. 3C, 3D, Table I). Aside from the restriction triad positions, Arg^62^ (contacting TCR α-chain Asp^94^ and Gln^98^), Gln^72^ (contacting TCR β-chain Gln^53^, Thr^55^, and Gly^56^), and Gln^152^ (contacting TCR α-chain Arg^97^ and β-chain Thr^100^) were the other important MHC contact residues, making a further 24 Van der Waals (Vdw), 1 salt-bridge, and 3 hydrogen bonds with the 3B TCR (Supplemental Table III).

### The TI8 peptide lacks the canonical central bulge

**FIGURE 4.**
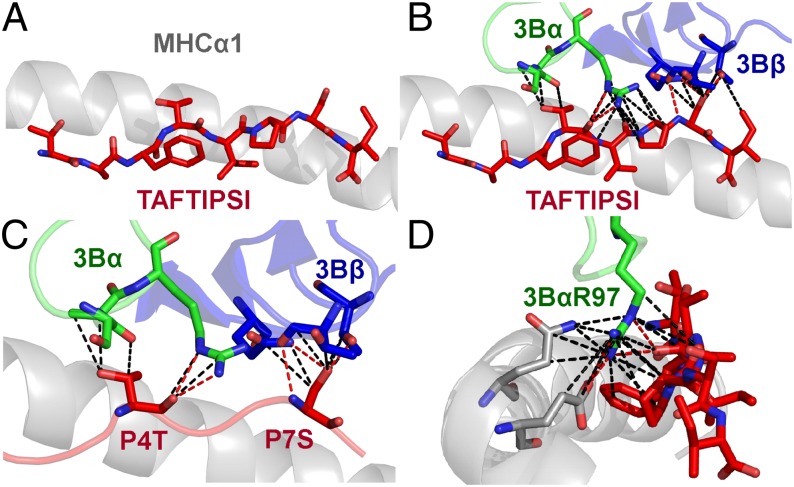
The TI8 peptide lacks the canonical central bulge. (**A**) Side view of the TI8 peptide (red sticks) conformation demonstrating the lack of a central peptide bulge. (**B**) Contacts between the 3B TCR (α in green sticks and β in blue sticks) and the TI8 peptide (red sticks). (**C**) Contacts between the 3B TCR (α in green sticks and β in blue sticks) and the peptide residues P4T and P7S (red sticks) that represent the majority of interactions between the TCR and peptide. (**D**) Contacts between the 3B TCR CDR3α-chain residue Arg^97^ and the B51 surface (gray sticks) and the TI8 peptide (red sticks). 3B TCR CDR3α-chain residue Arg^97^ interacts with a pocket formed by B51-TI8 in a ball-and-socket–like manner enabling a number of Vdw contacts (black dotted lines, 4Å cutoff) and hydrogen bonds (red dotted lines, 3.4 Å cutoff) to form at the TCR–pMHC interface.

Previous pMHCI structures have shown that the central residues in the peptide generally bulge out of the MHC binding groove mediating TCR contacts (25). The fixed anchors at the N and C termini of the peptide and the closed conformation of the MHCI groove force longer peptides to bulge further out of the groove to accommodate the extra central peptide residues (26). Different T cells have been shown to have a preference for peptides of specific length, presumably dependent on the size of the peptide bulge (28). The 3B–B51-TI8 complex solved in this study is the first ever, to our knowledge, human TCR–pMHCI complex with an 8-mer peptide. The TI8 peptide, being an 8-mer (the shortest length reported for natural MHCI binding peptides) lacked any prominent central bulge and was almost flat in the binding groove, akin to MHC class II peptide presentation (29, 30) (Fig. 4A). The consequences of this flat binding mode were 2-fold. First, the 3B TCR made only 33 contacts with the peptide (Table I), fewer than typically observed for other TCR-pMHCI structures (25). Usually the number of peptide contacts account for ∼35% of total contacts rather than the ∼25% as seen in this study. Second, the lack of peptide bulge enabled the TCR to form a closer interaction with the MHC surface, supported by the comparatively large BSA of 2041.2 Å observed for the TCR–MHC interface (Table I) (22). These observations demonstrate how the length of the peptide can govern pMHC interactions. For instance, a previously reported TCR–pMHC structure with a superbulged 13-mer peptide showed that the TCR perched on the extended peptide bulge and made limited contacts with the MHC surface (26).

### Peptide positions 4 and 7 are the main contact residues for the 3B TCR

The 3B TCR contacted six peptide residues in the B51-TI8 complex, with the TCRα-chain contacting peptide residues 3–6 and the TCRβ-chain contacting peptide residues 7 and 8. Despite this broad binding, a total of just 33 interactions were made with the peptide (Fig. 4B) compared with 123 with the MHC, illustrating the MHC-centric nature of the 3B–B51-TI8 interface. The side chains of peptide residues Thr^4^ and Ser^7^ were orientated up out of the groove and made the vast majority of the contacts, making all 5 of the hydrogen bonds and 17 of the 28 Vdw interactions between the 3B TCR and the peptide (Fig. 4C). The position of the 3B TCR during binding also demonstrated that, even though there was an amino acid difference (Glu-Lys) at position 104 in CDR3β of TRAV17/TRBV7-3 TCR in KI-112, KI-127, and KI-250, compared with that of the 3B TCR identified in CTL from KI-021 and KI-051, these differences were away from the binding interface of the TCR. Thus, assuming that these two TCRs use a similar binding register, which is likely as they only differ at one residue, they probably use the same molecular mechanism to engage B51-TI8. These data indicate that functionally identical TRAV17/TRBV7-3TCRs were selected in five out of seven HLA-B51:01^+^HIV^+^ patients.

### A conserved binding motif explains public selection of B51-TI8–specific TCRs

Although the interaction between the 3B TCR and B51-TI8 involved >20 different TCR residues (Supplemental Table III), TCR CDR3α residue Arg^97^ accounted for 3 out of 5 hydrogen bonds and 9 out of 23 Vdw contacts with the peptide and 3 out of 13 hydrogen bonds and 9 out of 110 Vdw contacts with the MHC surface (Fig. 4D). Arg^97^ made this contact network by fitting into a pocket formed by the MHC α2-domain and the peptide backbone, akin to a ball-and-socket joint. Thus, Arg^97^ is likely to be an important driving force in this otherwise weak TCR–pMHCI interaction, acting as an anchor grasping onto both the peptide and MHC. In support of this notion, Arg^97^ was conserved in the CDR3α loops of all of the TRAV17/TRBV7-3 TCRs detected (Fig. 1A), including from patient KI-391 that expressed a distinct CDR3α loop and ex vivo B51-TI8–specific CD8^+^ T cells in KI-051 (Fig. 1B, 1C). In addition to the dominant role for Arg^97^ in the CDR3α loop, the 3B TCR used a very broad binding motif that included the majority of residues in the TRAV17/TRBV7-3–encoded CDR1 and 2 loops. This additional network of interactions provided a stable binding platform, enabling Arg^97^ to perform its central role and thereby explaining the conservation of this clonotype. Only the combination of the TRAV17 and TRBV7-3 genes encoded for the correct arrangement of residues capable of interacting with B51-TI8 in this mode, probably explaining the predominance of such clonotypes in the B51-TI8 response.

## Discussion

HLA-B*27, HLA-B*51, and HLA-B*57 are associated with better control of HIV infection (3–5). Previous studies with HLA-B*27 and HLA-B*57 have identified residue identical public TCR responses [defined as residue-identical receptors found across different individuals who share a common MHC (22, 31)] against HIV (32–35). For example, public TCR clonotypes were detected in HLA-B*27–restricted p24 Gag-derived KK10-specific (KRWIILGLNK; residues 263–272) CTLs (32, 36). KK10-specific CTLs, characterized by *TRBV4-3/TRBJ1-3* or *TRBV6-5/TRBJ1-1* gene expression, were found to be preferentially selected in vivo and shared between individuals. CTLs expressing this public clonotype exhibited high levels of TCR avidity and Ag sensitivity, enabling functional advantages and effective suppression of HIV-1 replication (32, 36).

We previously demonstrated that the magnitude of B51-TI8–specific CD8^+^ T cells was significantly correlated to low plasma viral load in chronically HIV-1–infected HLA-B*51:01^+^ Japanese hemophiliacs (7). No correlation was found between the magnitude of the CD8^+^ T cell response to three other dominant HLA-B51–restricted HIV-1 epitopes (7), suggesting that B51-TI8–specific CD8^+^ T cells might play an important role in the control of viral replication in these patients. In this study, we identified a public CTL clonotype, expressing TRAV17/TRBV7-3 TCR genes, deployed against the immunodominant B51-TI8 epitope. These data indicate that even CTLs having weak TCR affinity could play a central role in the control of HIV-1. The 3B–B51-TI8 structure, which to our knowledge is the first TCR–pMHCI complex structure with the B51 allele and first structure of a human 8-mer peptide TCR–pMHC complex, demonstrated that the conformation of the TI8 peptide was relatively flat compared with the classical prominent central peptide bulge observed for most other pMHCIs. This flat conformation enabled the 3B TCR to bind in an MHC-centric manner, broadly contacting the residues that form the restriction triad. Despite the broad binding mode, a conserved Arg at position 97 in the CDR3α loop dominated contacts and was central to binding. Arg^97^ was also present in patients that used a TCR with a different CDR3α loop, underscoring its central role in the interaction.

Garcia et al. (37) previously solved the crystal structure of the mouse 2C TCR in complex with H-2K^b^ bound to the 8-mer self-peptide, dEV8 (EQYKFYSV). Analogous to the 3B TCR interaction, the 2C TCR, in complex with H-2K^b^-dEV8, showed weak affinity (*K*_D_ ≈ 84.1 μM) (38), and the crystal structure of the TCR–pMHC complex revealed that 2C TCR bound in an MHC-centric manner (37). Furthermore, the relative flatness of the TI8 peptide in the binding groove resembles the flat conformation typically observed in pMHC class II (pMHCII) structures (29). Like most TCR–pMHCII complexes, the 3B TCR bound relatively weakly in comparison with the average TCR–pMHCI interaction (24). The structures of the 3B and 2C TCRs suggest a general MHC-centric mode of binding to relatively featureless peptides. The relatively low affinities of 3B and 2C TCRs for their cognate peptides make it tempting to speculate that high-affinity TCRs specific for flatter pMHC landscapes might be deleted during thymic selection because high-affinity MHC-centric TCR binding may increase the potential for cross-reactivity with self. Thus, the weak affinity of the 3B TCR may represent the best B51-TI8 specificity that can escape thymic deletion.

In conclusion, the structure of the 3B TCR in complex with B51-TI8 demonstrates the molecular basis for the selection of this public CTL clonotype in HLA-B*51:01^+^HIV^+^ patients by revealing that TCR residues involved in the majority of interactions with the B51-TI8 surface were only encoded by the TRAV17/TRBV7-3 TCR genes. This contact network enabled the conserved Arg^97^ residue in the CDR3α loop to make optimal contacts with B51-TI8 via a ball-and-socket–type interaction. It is also noteworthy that this to our knowledge first ever human TCR–pMHCI struc-ture with an 8-mer peptide, which binds flat within the MHC groove to provide a relatively featureless landscape, showed similarities to the 2C interaction with H-2K^b^–dEV8 and TCR–pMHCII complexes. All of these TCRs bind to their relatively "vanilla" cognate ligands with weak affinity compared with TCRs that bind to the contoured landscapes of bulging MHCI-restricted foreign peptides of nine or more amino acids in length. We therefore suggest that weak affinity might be a common feature of TCR binding to flat peptides.
